# Examining the Interaction Between Perceived Neighborhood Disorder, Positive Peers, and Self-Esteem on Adolescent Prosocial Behavior: A Study of Chinese Adolescents

**DOI:** 10.3390/bs15091146

**Published:** 2025-08-23

**Authors:** Min Zhao, Qiannan Jia, Caina Li

**Affiliations:** 1 Faculty of Education, Shaanxi Normal University, Xi’an 710062, China; zhaomin2022@snnu.edu.cn; 2 School of Elementary Education, Changji University, Changji 831100, China; 3 Department of Military Medical Psychology, Air Force Medical University, Xi’an 710043, China; sherlyj@163.com; 4 School of Psychology, Shaanxi Normal University, Xi’an 710061, China; 5 Shaanxi Provincial Key Research Center of Child Mental and Behavioral Health, Xi’an 710061, China

**Keywords:** prosocial behavior, positive peers, self-esteem, neighborhood disorder

## Abstract

This study conducts a cross-sectional analysis to examine the complex relationship between perceived neighborhood disorder and adolescent social behaviors, aiming to enhance our understanding of the psychological mechanisms underlying this relationship. Based on a robust sample of 4399 Chinese adolescent participants (*M*_age_ = 14.16, *SD* = 2.11), including 2112 females (48% of the sample), this study examines the combined impact of perceived neighborhood disorder and the presence of positive peer relationships on adolescent prosocial behavior. It examines the potential mediating role of self-esteem within this dynamic. The methodological approach combines parental assessments of neighborhood disorder with adolescents’ reports of positive peer interactions, self-esteem, and prosocial behavior. Findings show a significant negative correlation between perceived neighborhood disorder and adolescent prosocial behavior. Unexpectedly, positive peer presence fails to mitigate this adverse effect, amplifying it instead. Moreover, self-esteem serves as a mediator in the relationship between perceived neighborhood disorder and the impact of positive peers on prosocial behavior. The findings align with the Bioecological Framework and the Reverse Stress-Buffering Model, underscoring the importance of environmental interactions in shaping adolescent behavior.

## 1. Introduction

Positive Youth Development (PYD) marks a significant paradigm shift in the research on youth development. Rather than focusing solely on developmental issues, it highlights the importance of individual plasticity and growth potential (Lerner et al., 2005). Aligning with the PYD framework, many developmental psychologists have increasingly focused on fostering positive outcomes in adolescents. Notably, defined as voluntary actions intended to benefit others, prosocial behavior has garnered significant attention due to its myriad emotional, cognitive, and behavioral benefits. Positive outcomes of prosocial behavior include improved self-esteem, interpersonal relationships, and academic performance (Caprara et al., 2015; Eisenberg et al., 2015; Fu et al., 2017). This study investigates the environmental factors and mechanisms that promote or hinder prosocial behavior among adolescents. Gaining a comprehensive understanding of these factors deepens our knowledge of adolescent prosocial behavior and equips educators and policymakers with evidence-based insights. These insights can inform the design of effective interventions and policies that promote positive youth development.

Bronfenbrenner’s (1979) and Bronfenbrenner and Ceci’s (1994) social ecological model posits that adolescent development is shaped by multiple environmental factors—such as neighborhood context and peer relationships—through interrelated, interactive systems. Despite the critical roles of neighborhood and peer environments as distal and proximal socialization systems, surprisingly little research has examined their potential interactive effects on adolescent prosocial behavior. Furthermore, Social Cognitive Theory suggests that environmental influences on prosocial behavior often operate indirectly through socio-cognitive and socio-emotional mechanisms, such as self-esteem (Carlo et al., 2018; Eisenberg et al., 2006).

Building on this framework, the present study explores how neighborhood context and peer influence—key distal and proximal environmental factors—interact to shape adolescents’ prosocial behavior, with a particular focus on the mediating role of self-esteem.

### 1.1. Perceived Neighborhood Disorder and Prosocial Behavior

Perceived neighborhood disorder—a key indicator of neighborhood context—refers to residents’ perceptions of conditions that signal a breakdown in social control. These may include visible disturbances, litter, crime, vandalism, and interpersonal conflicts (Kim & Conley, 2011; Ross & Mirowsky, 1999). Extensive research indicates that adolescents living in high-disorder neighborhoods are less likely to engage in prosocial behavior (Romero et al., 2015; Schofield et al., 2012).

Social Identity Theory posits that group membership shapes individuals’ emotions, thoughts, and behaviors through their sense of self (Trepte & Loy, 2017). Building on this framework, scholars have introduced the “social curse” phenomenon, which suggests that adverse social environments—such as neighborhoods with high disorder—impede positive development, including prosocial behavior. This effect is attributed to chronic stress, limited social support, and the reinforcement of maladaptive social norms (Wakefield et al., 2019). Moreover, adolescents in highly disordered neighborhoods are more likely to experience severe negative influences, such as violence and crime. They face elevated stress levels, while their families often lack the resources to provide adequate support. Additionally, the prevalence of antisocial norms in such environments fosters hostile group behavior, thereby further hindering the development of prosocial attitudes and actions. Empirical studies support these claims, indicating that adolescents exposed to high levels of neighborhood disorder exhibit lower levels of prosocial behaviors (Romero et al., 2015; Schofield et al., 2012).

### 1.2. Positive Peer Influence and Prosocial Behavior

As proximal environmental factors, positive peers have considerable influence on adolescents’ prosocial behavior, particularly given the increasing impact of peer influence on adolescents during puberty (Brown & Anistranski, 2021; Eisenberg et al., 2006). Positive peers can be defined as individuals who aspire for higher education, exhibit academic diligence, readily offer assistance, and participate in positive behaviors (Fuligni et al., 2001; Walters, 2020). Social Learning Theory posits that adolescents imitate peers’ behaviors through observational learning, particularly when these behaviors lead to social rewards (Bandura, 1986). Thus, adolescents are more likely to engage in prosocial behavior when surrounded by peers who consistently model such actions (Carlo & Pierotti, 2020; Giletta et al., 2021). Empirical evidence supports this theory, showing that interventions involving positive peers or peer-delivered feedback strengthen adolescents’ prosocial behavior.

As indicated by the Bioecological Framework, perceived neighborhood disorder and positive peers not only directly affect adolescents but also interact to shape their cognitive and behavioral development. However, two opposing viewpoints exist regarding how these factors combine to influence adolescents’ prosocial behavior. First, the Stress-Buffering Model suggests that positive peers may mitigate the adverse effects of perceived neighborhood disorder (Rueger et al., 2016). Specifically, the favorable interpersonal relationships that adolescents form with positive peers can enhance their positive connections and trust in others, offering protection from adverse neighborhood disorders (Matlin et al., 2011). Moreover, neighborhood disorder often exposes adolescents to numerous settings of misbehavior. However, as a proximal factor, positive peers can display good modeling behavior while buffering the impact of exposure to negative neighborhood deviance (Telzer et al., 2015). Aligning with this theory, empirical research shows that affiliation with positive peers can buffer adolescents from the adverse effects of neighborhood disorder and reduce substance use (Mason et al., 2017). Second, according to the Reverse Stress-Buffering Model, the adverse consequences of neighborhood disorder on adolescent development cannot be buffered by positive peers. Instead, positive peers can exacerbate the negative impact of neighborhood disorder on adolescents (Rueger et al., 2016). Neighborhood disorder, in a broader context, may be a stressor shared by adolescents and their positive peers, thereby undermining the quality of social support provided by these peers to adolescents (Minh et al., 2017; Roosa et al., 2003). Moreover, the Bioecological Framework suggests that adolescent development suffers when interactions among environmental settings are conflictual rather than supportive (Bronfenbrenner & Morris, 2006). Thus, cognitive and behavioral adjustment declines when the adverse effects of neighborhood disorder conflict with the social support provided by positive peers. Some studies also report that adolescents in disadvantaged neighborhoods who receive high levels of peer support exhibit more antisocial behavior (Silver, 2000).

Building upon existing theories and research, this study examines the moderating role of positive peers in the relationship between perceived neighborhood disorder and adolescents’ prosocial behavior. Although both factors influence adolescent development, little attention has been given to their interactive effects on self-esteem and prosocial behavior. The primary objective is to determine whether positive peers buffer or amplify the impact of perceived neighborhood disorder on adolescents’ prosocial behavior. While we hypothesize that positive peers conditionally mitigate or intensify these effects, no specific pattern is proposed.

### 1.3. The Role of Self-Esteem

Although perceived neighborhood disorder and positive peer interactions can influence adolescent prosocial behavior, the specific developmental mechanisms behind this association remain unexplored. Social Cognitive Theory suggests that self-esteem is not only a socio-cognitive and socio-emotional factor, but it also serves as a bridge between social environments (e.g., perceived neighborhood disorder, positive peers) and adolescent prosocial behavior. Research suggests a significant positive relationship between self-esteem and prosocial behavior, particularly toward friends and family. This finding indicates that enhancing self-esteem can improve adolescents’ social and emotional capabilities, thereby encouraging prosocial behavior. This context provides additional empirical support regarding the mediating role of self-esteem (Carlo et al., 2018; Eisenberg et al., 2006; Zuffianò et al., 2014).

Self-esteem is defined as an individual’s assessment of themselves. Further, perceptions of neighborhood disorder deeply influence self-esteem. Past research continually suggests that high neighborhood disorder has a significantly negative impact on residents’ self-esteem, especially among women (Hill et al., 2013). Recent studies suggest that living in disadvantaged neighborhoods, marked by high disorder, lowers self-esteem; however, social support can buffer this effect (Kim & Pai, 2022). Positive peer relationships, a key source of social support, may also protect adolescents by mitigating the negative impact of neighborhood disorder on their self-esteem (Pederson et al., 2022).

Interacting with positive peers provides a sense of safety and stability while fostering self-discovery and an understanding of others among adolescents. Such interactions help improve self-esteem and encourage prosocial behavior (Magro et al., 2019; Gorrese & Ruggieri, 2013; Spilt et al., 2014; Verschueren et al., 2012). Thus, adolescents with higher self-esteem tend to exhibit greater prosocial behavior. Against this backdrop, the second objective of this study is to investigate how self-esteem mediates the moderating effect of positive peers on the relationship between perceived neighborhood disorder and adolescent prosocial behavior.

To achieve this goal, this study adopted a survey methodology. We developed a structured questionnaire including dimensions of perceived neighborhood environment, peer relationships, self-esteem, and prosocial behavior. To collect their subjective assessments of these variables, the questionnaire was distributed to adolescents recruited through schools. For data analysis, we employed Structural Equation Modeling (SEM) to comprehensively investigate the mediating role of self-esteem in the relationship between perceived neighborhood disorder and prosocial behavior, as well as the moderating role of positive peer relationships.

### 1.4. The Current Study

Although existing research examines various factors that influence adolescent social behavior, it often overlooks the impact of community environments and peer relationships on internal psychological processes. In China’s unique social and cultural context, studies on the interaction between perceived neighborhood disorder and positive peer influence on adolescent prosocial behavior are limited. Using the Bioecological Model as a theoretical framework, this study analyzes data from 4399 Chinese adolescents to explore these complex relationships.

The significance of this study lies in examining the impact of the community environment on adolescent development while considering peer relationships as a moderator and self-esteem as a mediator. The findings enhance the understanding of adolescents’ psychological adjustments to community challenges, providing both theoretical and empirical support for designing more effective youth development programs.

Based on the above, this study proposes the following hypotheses:

**Hypothesis** **1.** *Positive peers moderate the relationship between perceived neighborhood disorder and adolescent prosocial behavior. In other words, adolescents supported by positive peers may exhibit more prosocial behaviors even in adverse neighborhood environments.*

**Hypothesis** **2.** *Adolescents’ self-esteem mediates the moderating effect of positive peers on the relationship between perceived neighborhood disorder and prosocial behavior, thus highlighting self-esteem as a key link between environmental factors and behavioral outcomes.*

By exploring these hypotheses, this study contributes new theoretical perspectives to the research on adolescent social behavior and provides data support for policy-making and practical applications. The contributions enrich the application of the Bioecological Model and elicit new insights into the field of social psychology.

## 2. Method

### 2.1. Participants and Procedures

Participants were recruited using a stratified cluster sampling method from six junior middle schools in Xi’an, China, covering both urban and suburban areas to ensure the representativeness of the sample. Due to practical constraints related to school accessibility, the sample was non-randomized. A total of 4399 adolescents (*M*_age_ = 14.16; *SD* = 2.11; 2112 females, 48% of the sample) participated in this study. A total of 98% of the participants identified as being of Chinese Han ethnicity. A total of 22.6% of fathers and 22.1% of mothers had a bachelor’s degree or above. All participants were Chinese-speaking.

First, all data collection procedures were approved by the Institutional Review Board at X University; informed consent was obtained from participants, their parents, teachers, and school principals before data collection. Next, research assistants presented information regarding the research plan and provided instructions on how to complete the questionnaires. Subsequently, participants all completed the online survey in their classroom during a class session. The procedure lasted approximately 30 min, and each student received a gift as compensation. All investigation procedures complied with ethical standards for scientific research.

### 2.2. Measures

#### 2.2.1. Perceived Neighborhood Disorder

The Perceived Neighborhood Disorder Scale (PNDS), reported by parents, assesses the neighborhood environment in our study (Ross & Mirowsky, 1999). The original scale comprises 15 items, rated on a four-point Likert scale, ranging from 1 (strongly disagree) to 4 (strongly agree). We adapted five items from the PNDS to measure parents’ perceptions of neighborhood disorder: “There are too many people hanging around on the streets near my home”, “My neighborhood is clean”, “My neighborhood is noisy”, “My neighborhood is safe”, and “I’m always having trouble with my neighbors”. The reliability of this adapted scale was acceptable, with Cronbach’s α = 0.61 in this study.

#### 2.2.2. Positive Peer

This study measured positive peer groups using the Positive Peer and Deviant Peer Questionnaire (Fuligni et al., 2001), containing seven items divided into two subscales: positive peers (e.g., “How many of your friends study hard each day”) and deviant peers (e.g., “How many of your friends punch or push another student”). Participants responded to all items on a five-point scale ranging from 1 (never) to 5 (always); Cronbach’s α = 0.74 for the positive peer subscale in the present study.

#### 2.2.3. Self-Esteem

We measured self-esteem using the Rosenberg Self-Esteem Questionnaire (RSES) (Rosenberg, 1965), which is a 10-item self-report measure with a five-point scale, ranging from 1 (strongly disagree) to 5 (strongly agree). The questionnaire measures positive and negative self-perceptions (e.g., “I consider myself a valuable person” and “All in all, I am inclined to feel that I am a failure”). Scores were averaged, with higher scores indicating higher self-esteem. In this study, Cronbach’s α = 0.70.

#### 2.2.4. Prosocial Behavior

We used a modified version of the Prosocial Tendencies Measure (PTM) to measure adolescents’ prosocial behavior (Carlo & Randall, 2002; Kou et al., 2007). The questionnaire comprises 26 items, each rated on a five-point scale ranging from 1 (does not describe me at all) to 5 (describes me greatly): public (e.g., “I prefer to help others in many public places”), anonymous (e.g., “I prefer to donate anonymously”), dire (e.g., “I tend to help people who are in real crisis or need”), emotional (e.g., “I respond to helping others best when the situation is highly emotional”), compliant (e.g., “When people ask me to help them, I don’t hesitate”), and altruistic (e.g., “I often help even if I don’t think I will get anything out of helping”). The internal consistency of this measure in our study was excellent, with Cronbach’s α = 0.96.

#### 2.2.5. Control Variables

We measured adolescents’ gender, age, subjective family economic status (SES), parents’ education level, and ethnicity. Subjective family economic status was measured as adolescents’ perceived family economic level, ranging from 1 to 10.

### 2.3. Data Analysis

**Figure 1:**
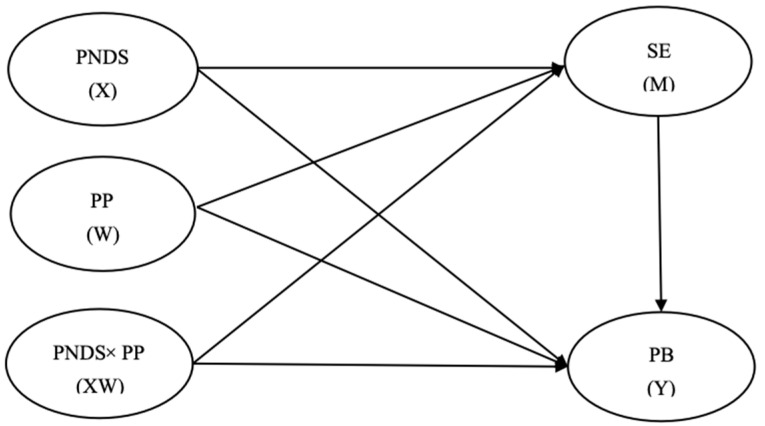
Proposed moderated mediation model. Note: PNDS = perceived neighborhood disorder; PP = positive peers; SE = self-esteem; PB = prosocial behavior.

This study used SPSS 22.0 to calculate descriptive statistics and to analyze the correlations between variables. Next, we used Mplus 8.3 to examine the hypothetical model (see Figure 1). First, the measurement model was evaluated using confirmatory factor analysis (CFA) to assess the validity and reliability of the latent constructs. Second, a moderation model was tested via the bootstrap method to examine the direct effects of perceived neighborhood disorder and positive peer relations, as well as their interaction, on prosocial behavior. Finally, Latent Moderated Structural Equations (LMS) were used to test the moderated mediation model, assessing both the mediating role of self-esteem in the path from perceived neighborhood disorder to prosocial behavior and the moderating role of positive peer relations in the paths from perceived neighborhood disorder to self-esteem and to prosocial behavior.

This study conducted Harman’s one-factor test to assess potential common method bias using factor analysis. The results reveal eight eigenvalues greater than one, with the first factor explaining 33.5% of the total variance across questionnaire items. This finding suggests that no significant common method bias was present in the current study, thereby ensuring the reliability and validity of the findings.

## 3. Results

### 3.1. Preliminary Analyses

Table 1 presents descriptive statistics and bivariate correlations for all variables in this study. As expected, perceived neighborhood disorder was negatively associated with positive peers, self-esteem, and prosocial behavior. Furthermore, the positive peer variable was positively associated with self-esteem and prosocial behavior. Self-esteem was also positively related to prosocial behavior.

### 3.2. Measurement Model Testing

CFA using FLML was performed to test the measurement model; four latent variables in the present study included perceived neighborhood disorder (X1, X2, X3, X4, X5), positive peers (PP1, PP2, PP3), self-esteem (M1, M2, M3), prosocial behavior (Y1, Y2, Y3, Y4, Y5, Y6). The results indicate that the measurement model fit the data perfectly: CFI = 0.97, TLI = 0.96, RMSEA = 0.05, and SRMR = 0.04. Unstandardized and standardized factor loadings for all variables are presented in Table 2.

### 3.3. Testing for the Moderating Effect of Positive Peers

After controlling for the adolescents’ gender, age, and subjective family economic status, Mplus 8.3 was used to perform LMS analysis to examine whether positive peers moderate the relationship between perceived neighborhood disorder and prosocial behavior. First, the basic model without the latent interaction (Model 1) showed LL = –70,592.061 and AIC = 141,304.122. Next, the model including the latent interaction (perceived neighborhood disorder × positive peers; Model 2) showed LL = –70,414.498 and AIC = 140,952.996. The difference, D = AIC(Model 1) − AIC(Model 2) = 355.13, with df = 2 and *p* < 0.001, indicates that the latent interaction significantly improves the model fit, confirming Model 2 as a suitable model.

**Figure 2:**
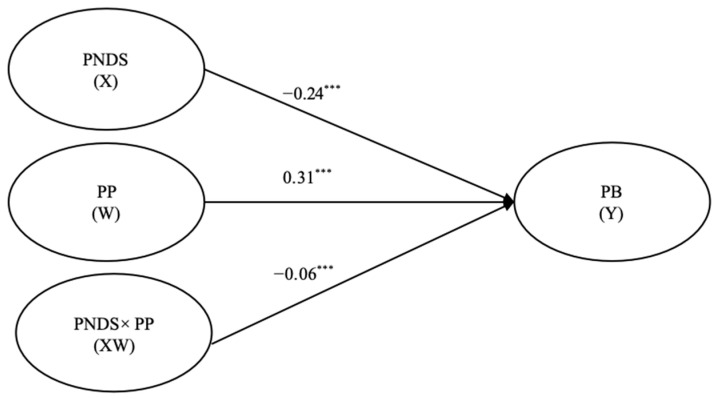
Moderated relationship model. Note: PNDS = perceived neighborhood disorder; PP = positive peers; SE = self-esteem; PB = prosocial behavior; *** *p* < 0.001.

Figure 2 shows that both perceived neighborhood disorder (*β* = −0.24, *p* < 0.001, 95% CI = [−0.27, −0.19]) and positive peers (*β* = 0.30, *p* < 0.001, 95% CI = [0.27, 0.34]) had significant effects on adolescent prosocial behavior. The latent interaction effect on prosocial behavior also was significant (*β* = −0.06, *p* = 0.001, 95% CI = [−0.26, −0.04]).

**Figure 3:**
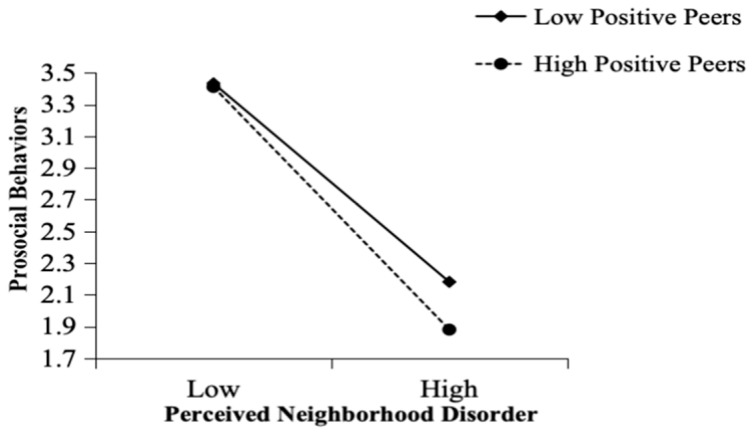
Moderating effect of positive peers on the relationship between perceived neighborhood disorder and prosocial behavior.

Furthermore, the significant interaction between perceived neighborhood disorder and positive peers was examined using a simple slope test. As shown in Figure 3, the moderating effect was evident under both high (*M* + 1*SD*) and low (*M* − 1*SD*) positive peer conditions. Regarding adolescents with fewer positive peers (*M* − 1SD), the relationship between perceived neighborhood disorder and prosocial behavior was weaker (B = −0.41, *p* < 0.001). In contrast, for adolescents with greater numbers of positive peers (*M* + 1*SD*), the relationship between perceived neighborhood disorder and prosocial behavior was stronger (B = −0.84, *p* < 0.001).

### 3.4. Testing for Mediated Moderation

**Figure 4:**
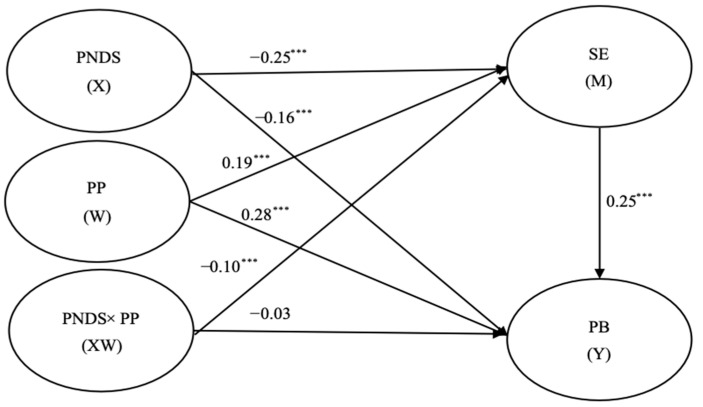
Mediated moderation model. Note: PNDS = perceived neighborhood disorder; PP = positive peer; SE = self-esteem; PB = prosocial behavior; *** *p* < 0.001.

Self-esteem was included as a mediating variable to build a mediated moderation model, which demonstrated acceptable fit. As shown in Figure 4, perceived neighborhood disorder (*β* = –0.25, *p* < 0.001, 95% CI = [−0.29, −0.21]), positive peers (*β* = 0.19, *p* < 0.001, 95% CI = [0.15, 0.22]), and the latent interaction effect (*β* = −0.10, *p* < 0.001, 95% CI = [−0.14, −0.06]) are all significantly associated with self-esteem. Self-esteem also positively mediated the relationship between the latent interaction and prosocial behavior (*β* = 0.25, *p* < 0.001, 95% CI = [0.22, 0.28]). After including self-esteem as a mediator, the direct effect of the latent interaction on prosocial behavior was no longer significant (*β* = −0.03, *p* = 0.12, 95%CI = [−0.07, 0.008]). This result suggests that self-esteem fully mediates the interaction.

**Figure 5:**
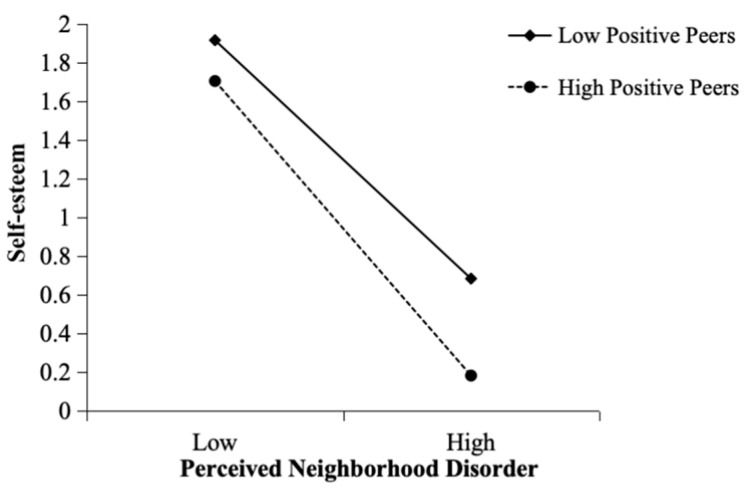
Moderating effect of positive peers on the relationship between perceived neighborhood disorder and self-esteem.

Figure 5 presents the results of the simple slope test: the moderating effect on the relationship between perceived neighborhood disorder and self-esteem is significant under both high and low positive peer conditions. For adolescents with fewer positive peers (*M* − 1*SD*), the moderated effect had a comparatively lower negative impact on self-esteem (B = −0.09, *p* < 0.001). In contrast, for adolescents with greater numbers of positive peers (*M* + 1*SD*), the moderated effect has a higher negative impact on self-esteem (B = −0.20, *p* < 0.001). The results emphasize the varying impact of positive peers on the relationship between perceived neighborhood disorder and adolescents’ self-esteem, underscoring the complexity of this interaction.

## 4. Discussion

Our study augments the theoretical framework by extending the Reverse Stress-Buffering Model (Rueger et al., 2016) within the context of Chinese adolescents’ prosocial behavior. While prior research typically supports the buffering role of positive peers in negative environments, our findings reveal a paradox: positive peers amplify—rather than mitigate—the adverse effects of perceived neighborhood disorder. This unexpected finding demands a reconsideration of the functions of protective factors in highly disordered settings, suggesting a complex dynamic where positive peers may heighten feelings of disparity and conflict instead of support.

By aligning the findings with the Bioecological Framework (Bronfenbrenner, 1979), we emphasize the complex relationship between multiple environmental factors and their collective impact on adolescent development. Our study highlights the importance of considering both microsystem and macrosystem influences, providing a nuanced understanding of adolescent behavior within socio-ecological contexts.

### 4.1. Moderating Role of Positive Peers

Aligning with Hypothesis 1, the present study found that positive peer moderates the relationship between perceived neighborhood disorder and adolescent prosocial behavior. Notably, positive peer—typically viewed as a protective factor—did not buffer but instead exacerbated the harmful effects of neighborhood disorder, supporting the Reverse Stress-Buffering Model rather than the Stress-Buffering Model (Rueger et al., 2016). Similar to the finding that fathers’ involvement fails to offset the impact of neighborhood disorder on child mental health, our results extend this pattern to peer relationships, showing that positive peers likewise cannot alleviate these adverse effects.

This finding extends the healthy context paradox, which indicates that a healthy school environment can lead to more severe maladjustment among bullied adolescents (Li et al., 2022). The present study extends this context to the interaction between community context and positive peers on adolescent prosocial behavior: adolescents living in high community disorder exhibit poor behavioral outcomes when they have greater positive peer.

This effect may stem from social comparison and performance pressure. In high-disorder neighborhoods, adolescents with more positive peers may engage in upward comparisons, becoming more aware of their environmental disadvantages, which could evoke feelings of relative deprivation and undermine self-esteem. In the Chinese cultural context—where harmony, peer norms, and mutual expectations are highly valued—positive peers may not only provide support but also impose implicit pressures to “live up to” their standards. This role strain may turn peer support into a double-edged sword, amplifying rather than buffering the negative impact of adverse community conditions on prosocial behavior.

### 4.2. Mediating Role of Self-Esteem

Confirming Hypothesis 2, this study found that the moderating effect of positive peers on the relationship between perceived neighborhood disorder and adolescent prosocial behavior operates through self-esteem. This finding aligns with Social Cognitive Theory, which posits that social environments shape individuals via cognitive mechanisms (Bandura, 1986; Carlo et al., 2018; Eisenberg et al., 2006). A possible explanation is that adolescents in highly disordered neighborhoods, even with positive peers, may develop negative self-concepts through upward social comparisons, reducing self-esteem (Hu et al., 2023). They may also lack interpersonal skills, which can lead to conflict in peer interactions and further diminish their self-esteem (Perez et al., 2001). Consequently, adolescents with lower self-esteem tend to lack confidence in their abilities, which hinders their engagement with prosocial behavior (Johnson, 2011; Krause et al., 2016). These findings highlight the complex interplay among neighborhood context, peer influence, self-esteem, and prosocial behavior, underscoring the need for interventions that strengthen self-esteem to promote positive social development.

### 4.3. Limitations and Future Research Directions

Several limitations of this study warrant caution when interpreting the findings, suggesting directions for future research. First, although our cross-sectional survey used a large sample (N = 4399) to examine relationships among variables, causal directions remain uncertain. A three-wave longitudinal design could help clarify these relationships.

Second, this study did not examine the role of group norms in shaping prosocial behavior, which may vary across different social levels—such as peer circles, classrooms, or schools—and influence how adolescents perceive and respond to social contexts. The absence of multilevel data limits our ability to explore how normative climates at higher ecological levels may shape individual prosocial tendencies. Future research should investigate these contextual influences using hierarchical designs to better understand the social dynamics involved.

Third, this study utilized a revised version of the Perceived Neighborhood Disorder Scale, which may have compromised assessment accuracy. Although the revised scale was tailored to Chinese culture, future research should explore the use of more reliable scales to enhance measurement precision in assessing perceived neighborhood disorder.

Finally, the sample was drawn from a single city (Xi’an) and is ethnically homogeneous, which may limit the generalizability of the findings. Future studies should replicate this model in more diverse cultural and regional contexts to enhance its external validity.

## 5. Strength and Implications

One key strength of this study is its large sample size (N = 4339), which allowed us to examine the interactive effects of multiple environmental factors on adolescents’ prosocial behavior. Additionally, the findings provide valuable insights for youth development initiatives.

First, the findings of this study indicate that positive peer are unable to mitigate the harmful effects of perceived neighborhood disorder on adolescent cognition and behavior. These findings advance the Reverse Stress-Buffering Model and deepen understanding of the healthy context paradox. Consequently, interventions for adolescents in highly disordered neighborhoods should integrate multiple protective factors rather than relying on peers alone.

Second, this study identified adolescent self-esteem as a key mechanism linking the interaction between perceived neighborhood disorder and positive peers to adolescents’ prosocial behavior. These findings underscore the substantial impact of multiple environmental factors and cognitive influences on the development of prosocial behaviors in adolescents. For example, for schools located in high-disorder neighborhoods, we recommend implementing weekly self-esteem workshops, such as strength-identification activities, to enhance adolescents’ self-worth. Additionally, training programs for positive peers should be conducted to help them avoid behaviors that may exacerbate status comparisons among peers. For community organizations, joint clean-up campaigns and safety patrols involving adolescents can be organized to improve neighborhood order, creating a more supportive environment for the development of prosocial behavior. Implementing these strategies not only addresses the impact of community disorder on adolescent behavior but also enhances their overall well-being and development in complex social contexts.

## 6. Conclusions

This study extends the Bioecological Framework and the Reverse Stress-Buffering Model through a cross-sectional survey design. We examined the association between perceived neighborhood disorder and positive peer relationships and adolescent prosocial behavior. Moreover, we explored the role of self-esteem in mediating the relationship between positive peer relationships and prosocial behavior. The findings indicate that perceived neighborhood disorder and positive peer relationships interact significantly: positive peers may exacerbate, rather than buffer, the negative impact of perceived neighborhood disorder on adolescent prosocial behavior.

Moreover, adolescent self-esteem acts as a mediator between the interactive effects and prosocial behavior. The findings underscore the critical importance of understanding the interaction between distal factors, such as neighborhood disorder, and proximal factors, including positive peer, in shaping adolescent prosocial behavior. A negative neighborhood context can negate the positive impact of other supportive environments on adolescents. Therefore, identifying adolescents in adverse neighborhood contexts and protecting them from the harmful effects of these environments is essential. This awareness is necessary for designing targeted interventions and support systems to promote positive prosocial behaviors among adolescents, particularly those living in challenging neighborhood circumstances.

## Figures and Tables

**Table 1:** Descriptive statistics and bivariate correlations for all variables.

	*M*	*SD*	1	2	3	4	5	6	7
1 Age	14.16	2.2	1						
2 Gender	-	-	−0.02	1					
3 SES	5.16	1.63	−0.13 **	0.2	1				
4 PP	3.04	0.59	−0.02	0.1 **	0.07 **	1			
5 SE	3.30	0.57	0.14	−0.05 **	0.1 **	0.18 **	1		
6 PB	3.93	0.66	−0.11 **	0.01	0.47 **	0.30 **	0.30 **	1	
7 PNDS	1.89	0.54	0.1	−0.03 *	−0.09 **	−0.22 **	−0.24 **	−0.24 **	1

Note: Gender was a dummy variable coded as 2 = female and 1 = male; SES = subjective family economic status; PNDS = perceived neighborhood disorder; PP = positive peer; SE = self-esteem; PB = prosocial behavior; * *p* < 0.05, ** *p* < 0.01.

**Table 2:** The CFA results for all variables.

Variable	Unstandardized Factor Loading	*SE*	*t*	Standardized Factor Loading
PNDS				
X1	1.00	0.00	999.00	0.44
X2	0.77	0.07	10.61	0.35
X3	1.04	0.05	18.95	0.48
X4	0.81	0.07	11.42	0.37
X5	1.02	0.06	15.37	0.50
PP				
PP1	1.00	0.00	999.00	0.76
PP2	1.45	0.03	36.80	0.81
PP3	0.73	0.02	30.66	0.53
SE				
M1	1.00	0.00	999.00	0.80
M2	0.98	0.02	43.61	0.86
M3	0.39	0.01	35.95	0.57
PTM				
Y1	1.00	0.00	999.00	0.88
Y2	1.02	0.01	91.58	0.92
Y3	1.05	0.01	83.78	0.89
Y4	1.04	0.01	77.06	0.85
Y5	0.94	0.01	80.19	0.79
Y6	0.93	0.01	75.03	0.84

Note: PNDS = perceived neighborhood disorder; PP = positive peer; SE = self-esteem; PB = prosocial behavior.

## Data Availability

The data presented in this study are available on requestfrom the corresponding author.
